# The relationship between BMI and physical fitness among 7451 college freshmen: a cross-sectional study in Beijing, China

**DOI:** 10.3389/fphys.2024.1435157

**Published:** 2024-10-15

**Authors:** Tongtong Guo, Siqin Shen, Sanjun Yang, Fan Yang

**Affiliations:** ^1^ Department of Physical Education and Research, China University of Mining and Technology-Beijing, Beijing, China; ^2^ Department of Physical Education, Second Affiliated Middle School of China People’s University, Beijing, China; ^3^ College of Physical Education, Dalian University, Dalian, China; ^4^ Li Ning Sports Science Research Center, Li Ning (China) Sports Goods Company Limited, Beijing, China

**Keywords:** physical fitness index, college freshmen, BMI levels, physical education, physical activity

## Abstract

**Objective:**

To identify trends in physical fitness test scores of college freshmen and their physical fitness from 2018 to 2021, and to analyze the relationship between college students’ Body Mass Index (BMI) and Physical Fitness Index (PFI).

**Methods:**

This study obtained physical fitness test data from 7,541 freshmen at a university in Beijing, China, from 2018 to 2021. Analysis of variance (ANOVA) was used to compare the physical fitness indicators among different BMI levels by gender. A nonlinear quadratic regression model was used to evaluate the relationship between BMI and each indicator within gender groups.

**Results:**

The BMI of freshmen in China was generally increased over the study period, and BMI levels influenced students’ physical fitness indexes to varying degrees. BMI was significantly correlated with the physical fitness indexes and PFI. The increase in BMI had a greater influence on the PFI of males than females.

**Conclusion:**

Students with a normal BMI show better physical fitness. A BMI below or above the normal range may result in poor physical fitness. The relationship between BMI and PFI has an inverted u-shaped curve. Physical education programs should be tailored to students with different fitness levels and fundamentals, including but not limited to the development of strength, speed, and other qualities.

## 1 Introduction

Physical fitness is a crucial component of students’ healthy physical and mental development. Physical fitness indicators serve as critical markers that reflect various physical attributes of individuals (Zhang et al., 2021), and these include the standing long jump, pull-up, sit-and-reach, 50 m sprint, endurance run and more. Some studies have indicated that greater baseline levels of cardiorespiratory fitness and muscle strength, which represent physical health (Zhang Y. et al., 2022), lead to better working memory performance and improved inhibitory control in children and adolescents (Godoy-Cumillaf et al., 2023; Zhao et al., 2021).

Body mass index (BMI) is a unified index for the global evaluation and monitoring of nutrition and weight status, and maintaining an ideal BMI is a guarantee of health and quality of life throughout the life cycle. In recent years, foreign scholars have pointed out that the relationship between insufficient physical activity and obesity is definite, and there is a dynamic relationship between obesity and four factors: physical activity, motor skill ability, perceptive motor ability (Barnett et al., 2008) and physical health (Stodden et al., 2008). Adolescents with excessive body fat are more likely to suffer from increased heart load, changes in lung function, endocrine disorders and immune disorders (Eckel et al., 2016).

Higher education is the last stage for teenagers to participate in school physical education, the transition stage for college students to the society, and the key period for developing healthy behaviors. College physical education bears the responsibility of improving college students’ physical health level and lifelong physical education ability. The first year is the first stage for college students to enter the university, and it is also a key period to improve physical fitness and develop lifelong sports habits (Dong et al., 2023). Changes in curriculum structure and lifestyle (Rafique et al., 2022) during college have led to an increase in sedentary behavior among freshmen (Hu et al., 2022). Sedentary behavior has become a major public health problem, accounting for 3.8% of all-cause mortality, and there is a strong correlation with obesity and cardiovascular disease (Li et al., 2008). At the same time, stress will also affect the quality of sleep (Li, 2022; Alkatan et al., 2021), which will further affect the metabolism of students (Fan et al., 2021). In addition, excessive intake of energy and daily junk food at night (Liu et al., 2021), coupled with the increasing proportion of lack of physical activity, will lead to the increase of students’ BMI and aggravate the risk of chronic diseases (Jha et al., 2021; Chen P. et al., 2020).

The Department of Physical Education, Health and Art Education of the Ministry of Education of China released the latest survey report on the Physical Health of Chinese College Students in 2020 (Department of Physical Health and Arts Education Ministry of Education, 2021). The report showed that the average comprehensive physical fitness score of college students was 71.23 points, marking a decrease of 2.18 points compared to the score in 2010. Notably, the failure rate of physical health was as high as 30.9%. Obesity and overweight persistent prevalent, affecting 23.8% of the student population. Furthermore, the Blue Book for Children 2021 underscores the severity of this issue (Yuan, 2021), reporting by 2022, the incidence of overweight and obesity among school-age children in China soared to 29.4%, and adolescent obesity was more likely persisting into adulthood (Beets and Pitetti, 2004). Moreover, the proportion of overweight and obesity among adults in China reached 50.7% by 2022 (Zhu and Yin, 2023), which not only increases the risk of disease, but also increases the obesity epidemic. It will also increase their psychological stress (Chew et al., 2022) and reduce their perception of happiness (Mo and Bai, 2023; Han et al., 2023).

However, due to the extremely high expectations of Chinese society and families for students’ academic performance, physical education has gradually been marginalized, which some scholars believe may be the reason for the declining health level of Chinese youth (Zhang X. et al., 2022). The physical functions of college students in terms of physical flexibility, cardiopulmonary endurance and so on are generally declining. Recent scholarship, both domestic and international, highlights the correlation between BMI and PFI with students’ physical health and sports literacy (Huang and Malina, 2007; Chen X. et al., 2020).

Motivated by these concerns, this study aims to explore the relationship between BMI and physical fitness of Chinese college students. To achieve this objective, we collected the national physical health test data from 7451 freshmen at a university in Haidian District, Beijing, China, spanning from 2018 to 2021. Although the data collection ended in 2021, the observed trends and relationships remain relevant today. The marginalization of physical health, physical activity and physical education has not yet changed substantially, and physical problems such as rising obesity rates and insufficient vital capacity remain shortcomings in the development of young people. Thus, it indicates that issues related to obesity and physical inactivity continue to be prevalent among college students (Han et al., 2023; Maitiniyazi et al., 2021; Sun et al., 2022), and highlights the ongoing need for effective physical education programs. But in existing studies, the correlation between an indicator and BMI is usually analyzed separately, and there are still fewer studies on the overall before and after comparisons during the epidemic. Therefore, we analyzed the correlation between BMI and physical fitness of college students in the period 2018 to 2021 to provide references for the physical and mental health as well as the all-round development of college students.

## 2 Materials and methods

### 2.1 Participants

This study is based on the physical fitness data of students enrolled in a selected university in Haidian District, Beijing, China. To enhance the reliability of our research findings and minimize potential fluctuations arising from single-year data, we opted to utilize physical fitness data spanning four consecutive years, from 2018 to 2021. Our study adhered to the standard assessment protocol outlined in the National Student Physical Fitness Standard of China (version 2014) (Ministry of Education of the People’s Republic of China, 2014).

Physical fitness tests are administered by physical education teachers during the first to third weeks of November of each school year and assessed by the same teachers and post-training assistants at the same location on campus during normal school hours, ensuring uniformity and reliability.

The comprehensive fitness program, as per the2014 edition of the standards, encompassed a range of assessments, including measurements of BMI, vital capacity, 50 m sprint, standing long jump, Sit-and-reach, endurance running (1000-m run for males and 800-m run for females) and sit-up (females) and pull-up (males). The number of assessments at each level is shown in Table 1.

**TABLE 1:** Assessment tasks, evaluation purposes and weights of the National Student Physical Fitness Standard of China (version 2014).

Gender	No.	Evaluation purposes	Task	Metric	Methods	Weight
All Genders	5		BMI	kg/m^2^	BMI (body mass index) = weight (kg)/height (m2). Weight and height were measured by ultrasonic measuring instrument and weight scale, and the accuracy of height and weight was 0.01 cm and 0.1kg, respectively	15
		Cardiopulmonary function	Vital Capacity	mL	Vital Capacity is an important indicator of cardiopulmonary function. Before the test, students stand in a straight position, tilt their head slightly back for maximum inhalation, and then slowly exhale, repeat the measurement twice, and record the best score	15
		Speed quality and explosive power	50 m sprint	Time (seconds)	Assess the speed quality of students in seconds, measuring their speed and acceleration in a 50-m dash, accurate to 0.01 s	20
		Flexibility	Sit-and-reach	Max Reach distance (cm)	Designed to assess the students’ physical flexibility and flexibility. During the test, students sit with their torso leaned forward and their legs extended as far forward as possible. Repeat the measurement twice and record the best result	10
		Lower body’s explosive power	Standing long jump	Max Reach distance (cm)	Designed to assess students’ explosive power. Students stand on the test mat, feet naturally open shoulder width, after the jump line to do upright preparation, two feet in place at the same time to jump, repeat twice, record the best results	10
Male	7	Upper body’s muscular strength and core strength endurance	Pull-up	No. of reps	The aim was to assess the muscle endurance of male students by recording the number of pull-ups they completed. During the test, the students held their hands inverted, with their bodies suspended vertically and at rest, and then used their arms to pull their up, pausing for a moment when they exceeded the horizontal bar, followed by restoring and repeating	10
		Cardiorespiratory endurance	Endurance run (1000 m)	Time (seconds)	It is designed to assess the muscular and cardiorespiratory endurance of students. The time taken by male students to complete 1000 m was recorded in seconds	20
Female	7	Core strength endurance	1 min sit-up	No. of reps	The aim was to assess the muscle endurance of female students by recording the number of sit-up they completed in 1 min. Before the test, students lie on their back with knees bent 90° and fingers touching ears. During the test, the student raises his or her torso until the elbows touch the knees and then returns to the starting position	10
		Cardiorespiratory endurance	Endurance run (800 m)	Time (seconds)	It is designed to assess the muscular and cardiorespiratory endurance of students. The test is conducted in the same way as for male students	20

### 2.2 Assessment indicators and tasks

According to the National Student Physical Fitness Standard of China (version 2014), the comprehensive physical fitness score is calculated by weighted summation of 5–7 normalized indicators, as shown in Table 1 (Cole et al., 2005). The maximum overall fitness score is 100. In the early 1980s, the Chinese Sports Science Society pointed out in its definition of “physical health” that health needs include five aspects: physical development, physical function, physical fitness and athletic ability, psychological development and adaptive ability. The content of physical fitness test focuses more on physical development and function, physical fitness and athletic ability (Caspersen et al., 1974), This includes assessments related to: (a) Physical Development and Function: Vital capacity, body mass index (BMI) based primarily on height and weight (Cater, 1992). (b) Cardiorespiratory Endurance: Endurance running. (c) Muscle Endurance: Sit-up and pull-up. (d) Explosive Power: Standing long jump and 50 m sprint. (e) Flexibility: Sit-and-reach.

To compare the physical health of children and adolescents with different BMI levels, the BMI of male and female was divided into four percentile grades: low weight, normal weight, overweight and obesity. The range of BMI values for each group is shown in Table 2.

**TABLE 2:** Body Mass Index rating scale.

Levels	Score (points)	Male	Female
Normal	100	17.9–23.9	17.2–23.9
Low weight	80	≤17.8	≤17.1
Overweight	80	24.0–27.9	24.0–27.9
Obesity	60	≥28.0	≥28.0

Note: Body Mass Index (BMI) (unit: kg/m^2^).

In addition, the relationship between physical fitness index and BMI was also explored. The Z-score was calculated as

$$Z-\text{score}=\frac{\text{Test}\;\text{Value}-\text{Average}\;\text{Value}\;\text{of}\;\text{item}}{\text{Standard}\;\text{Deciation}\;\text{of}\;\text{item}}\;.$$

Physical Fitness Index (PFI) is the total value of Z-score obtained after data standardization of various physical fitness indicators in the measurement of students’ system (Bi et al., 2019). Because the shorter the time of running 50 m, 1000 m and 800 m, the better the performance, the Z-scores for 50 m sprint and endurance run were reversed. Therefore,

$$\text{PFI}=Z_{\text{lung}\;\text{capacity}}+Z_{\text{standing}\;\text{long}\;\text{jump}}+Z_{\text{sit}-\text{and}-\text{reach}}+Z_{\text{core}\;\text{strength}\;\left(\text{male}\;\text{pull}-\text{ups}/\text{female}\;1\;\min\;\text{sit}-\text{ups}\right)}-Z_{50m\;\text{sprint}}-Z_{\text{endurance}\;\text{run}\;\left(\text{male}\;1000m/\;\text{female}\;800m\right).}$$

### 2.3 Statistical analysis

Before the analysis, this study compiled the basic information of students in each assessment year, including the number of students and gender, and used the mean (standard deviation) format to display descriptive statistics including BMI, various health indicators and PFI to analyze the basic characteristics of the samples (see Table 3).

**TABLE 3:** Indicators of students of different genders in different years of enrollment.

Gender	Grade	*N*	BMI(kg/m^2^)	Vital Capacity (mL)	50 m Sprint(s)	Standing long jump (cm)	Sit and reach (cm)	Endurance running speed (m/s)	Coreforce score (points)
			Mean (SD)	Mean (SD)	Mean (SD)	Mean (SD)	Mean (SD)	Mean (SD)	Mean (SD)
Male	2018	975	22.16 (3.63)^ωφλ^	3881.75 (614.57)^ωφλ^	8.01 (0.49)^ωφλ^	218.62 (20.26)^λ^	16.12 (6.67)^ωφ^	4.05 (0.42)^ωφ^	17.00 (26.48)^ωφ^
	2019	1281	22.55 (3.94)^ω^	4034.79 (644.57)^ωεδ^	7.80 (0.54)^ωε^	218.81 (21.46)^δ^	13.54 (7.18)^ωδ^	3.95 (0.40)^ωεδ^	13.40 (22.64)^ω^
	2020	1269	22.86 (3.90)^φ^	4237.75 (691.03)^ωε^	7.92 (0.57)^φεγ^	217.28 (21.60)	13.44 (6.77)^φγ^	4.01 (0.43)^φεγ^	12.21 (23.26)^φγ^
	2021	1271	22.74 (4.07)^λ^	4200.36 (667.85)^λδ^	7.83 (0.59)^λγ^	216.80 (20.47)^λδ^	15.78 (6.89)^δγ^	4.05 (0.43)^δγ^	15.51 (25.97)^γ^
	Total	4796	22.60 (3.91)	4101.26 (671.23)	7.88 (0.56)	217.83 (21.01)	14.63 (7.00)	4.01 (0.44)	14.28 (24.58)
Female	2018	664	21.08 (2.87)	2629.52 (462.28)^ωφλ^	9.87 (0.65)^ωφλ^	162.98 (15.53)^ωφ^	19.13 (5.73)^ωφ^	3.32 (0.32)^ωφλ^	62.33 (14.83)^φλ^
	2019	664	20.94 (2.90)^δ^	2745.01 (455.40)^ωεδ^	9.70 (0.66)^ω^	166.79 (18.18)^ωδ^	18.00 (6.35)^ωεδ^	3.22 (0.28)^ωεδ^	61.43 (17.15)^εδ^
	2020	675	21.22 (3.26)	2910.73 (470.62)^φε^	9.73 (0.65)^φ^	167.14 (22.68)^φγ^	17.33 (5.88)^φεγ^	3.26 (0.29)^φε^	65.95 (13.66)^φε^
	2021	652	21.30 (3.22)^δ^	2883.96 (475.13)^λδ^	9.69 (0.67)^λ^	162.40 (17.18)^δγ^	18.94 (5.96)^δγ^	3.27 (0.32)^λδ^	64.51 (16.48)^λδ^
	Total	2655	21.13 (3.07)	2792.38 (479.20)	9.75 (0.66)	164.85 (18.72)	18.34 (6.03)	3.27 (0.30)	63.56 (15.67)
Total	2018	1639	21.72 (3.39)^ωφλ^	3374.44 (830.20)^ωφλ^	8.76 (1.08)^ωφλ^	196.08 (32.99)^ωφλ^	17.34 (6.47)^ωφλ^	3.76 (0.53)^ω^	-
	2019	1945	22.00 (3.70)^ωεδ^	3594.47 (847.65)^ωεδ^	8.45 (1.08)^ωε^	201.05 (32.01)^ωδ^	15.06 (7.22)^ωδ^	3.70 (0.50)^ωεδ^	-
	2020	1944	22.29 (3.77)^φε^	3776.98 (887.58)^φε^	8.55 (1.05)^φεγ^	199.87 (32.45)^φ^	14.79 (6.73)^φγ^	3.75 (0.53)^εγ^	-
	2021	1923	22.25 (3.86)^λδ^	3754.03 (871.62)^λδ^	8.46 (1.08)^λγ^	198.36 (32.25)^λδ^	16.85 (6.76)^λδ^	3.79 (0.54)^δγ^	-
	Total	7451	22.08 (3.70)	3634.87 (874.51)	8.55 (1.08)	198.95 (32.45)	15.95 (6.90)	3.75 (0.52)	-

Note: A: grade 2018; B: grade 2019; C: grade 2020; D: grade 2021; *p* < 0.05: A vs. B ω; A vs. C φ; A vs. D λ; B vs. C ε; B vs. D δ; C vs. D γ.

The basic levels of health indicators of different school years and gender groups were analyzed. Data were tested for normality using Q-Q plots, followed by a one-way ANOVA to compare the results of physical fitness indicators between students of different genders under different years of enrollment, a two-way ANOVA to compare the results of physical fitness indicators between genders under different BMI grades, followed by Bonferroni adjustments for subsequent pairwise comparisons. And its effect size (ES) was analyzed with Partial η^2^ (small ES: 0.01 ≤ η^2^ < 0.06; Medium ES: 0.06 ≤ η^2^ < 0.14; Large ES: η^2^ ≥ 0.14). Pearson correlation analysis was used to verify the correlation between continuous variables. Pearson correlation coefficient is explained as follows (absolute value of R) \: R (no correlation), 0 < R < 0.1 (very weak correlation), 0.1 ≤ R < 0.3 (weak correlation), 0.3 ≤ R < 0.5 (moderate correlation), 0.5 ≤ R < 0.7 (strong correlation), R ≥ 0.7 (very strong correlation).

In order to describe the nature of the relationship between BMI and health indicators of the gender group, a nonlinear quadratic regression model was used to evaluate the relationship. The regression analysis established Y = aX^2^ + bX + c (Y = Z-score, X = BMI), where a, b and c were constants.

The statistical significance level was set to 0.05, and all analyses were performed using SPSS 26.0 statistical software.

## 3 Results

### 3.1 Basic information of BMI and PFI of freshmen

Data were completed screened to remove student grades containing invalid data before descriptive analyses were conducted. Table 3 outlines the fundamental characteristics of the cohort of first-year students, differentiated by gender and year of enrolment. The average BMI of the 7451 freshmen was 22.08 (3.70), with normal weight rate, low weight rate, overweight rate and obesity rate being 69.57%, 6.4%, 16.82%, and 7.21%.

Among the 4,796 males students, the average BMI was 22.60 (SD = 3.91), with corresponding rates of 63.45% for normal weight, 7.51% for low weight, 19.60% for overweight, and 9.45% for obesity. Notably, the normal weight rate among females, comprising 2,655 students, was significantly higher at 80.64% than that of male. Male’s BMI showed statistical significance (*p* < 0.05), except for the variance between 2020 and 2021 freshmen, which exhibited significance (*p* < 0.05) between groups with a descending order of 2018, 2019, 2021, and 2020. In the females’ group, the difference between 2019 and 2021 was statistically significant (*p* < 0.05), and there was no significant difference among other groups with a descending order of 2019, 2018, 2020, and 2021 grade.

### 3.2 Correlation between freshmen performance of various indicators and BMI

The bivariate correlation analysis revealed that the correlation coefficient R between BMI and physical fitness indexes of males and females students ranged from 0.054 to 0.406, and all the samples were statistically significant (*p* < 0.01) due to the large sample sizes. According to Pearson’s correlation coefficient R, the 50 m sprint, 800 m run, and PFI of females showed weak correlations, whilst the sit and reach and sit-up showed very weak correlations. In males, the sit and reach showed a very weak correlation and PFI was weakly correlated, whilst the rest showed moderate correlations. There was a positive correlation between the various BMIs of male and female students, vital capacity, and sit and reach. However, there was a negative correlation with physical fitness performance in events such as 50 m sprint and endurance running, standing long jump, and pull-up/1 min sit-up and PFI (*p* < 0.01). The effect of BMI changes on male students’ physical fitness performance was more prominent than that of female students in all events (see Table 4).

**TABLE 4:** Correlation between BMI levels and physical fitness indexes of students of different genders (R-value).

Gender	N	Vital capacity	50 m Sprint	Standing long jump	Sit and reach	Endurance run 1000 m/800 m	Pull-up/1min sit-up	PFI
Male	4796	0.35^**^	0.33^**^	−0.33^**^	0.07^**^	0.41^**^	−0.30^**^	−0.29^**^
Female	2655	0.31^**^	0.14^**^	−0.14^**^	0.05^**^	0.23^**^	−0.05^**^	−0.10^**^

Note: ** At a significance level of 0.01 (two-tailed), the correlation was statistically significant.

### 3.3 Freshmen physical indicators

Table 3 makes descriptive statistics for the students’ physical fitness indicators and presents them in the format of Mean (SD), and also identifies the results of pairwise comparisons of students’ physical fitness indicators for different years of enrollment and genders in the form of superscripts in the table. Using one-way ANOVA to compare the physical fitness test scores of freshmen of all genders in different entry years, the results showed that the main effects were all significant (*p* < 0.05), and the differences between groups in the spirometry index (F = 100.92, *p* < 0.01) were statistically significant (*p* < 0.01) with the exception of the 2020 and 2021 class; the 50 m run (F = 35.09, *p* < 0.01) the differences between the groups were statistically significant (*p* < 0.01) with the exception of the 2019 and 2021 class; the standing long jump (F = 8.77, *p* < 0.01) the differences between the performance of the 2021 class and that of the 2018 and 2019 class were statistically significant (*p* < 0.01). The average speed of male and female students was the slowest in class of 2019 and the fastest in the class of 2021, and except for the results of the class of 18 and the class of 20, the rest of the groups were significantly different from each other (*p* < 0.05); the results of core strength endurance (F = 4.44, *p* < 0.05) were the best for the female students of the class of 2020, while the male students of the class of 2018 were the best.

**FIGURE 1:**
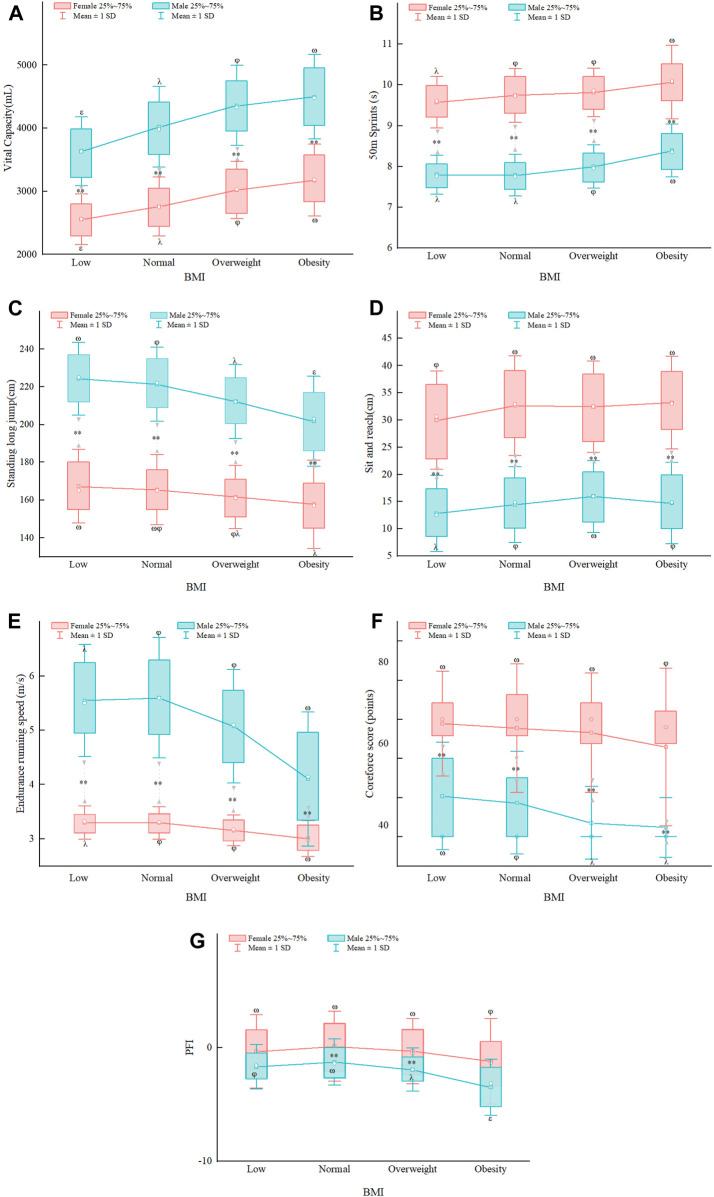
Plot of the effect of different BMI levels in various physical fitness indicators on freshmen of different genders. **(A)** The effect of different BMI levels on freshmen vital capacity; **(B)** The effect of different BMI levels on freshmen 50 m sprint; **(C)** The effect of different BMI levels on freshmen standing long jump; **(D)** The effect of different BMI levels on freshman sit and reach; **(E)** The effect of different BMI levels on freshmen endurance running speed; **(F)** The effect of different BMI levels on freshman coreforce score; **(G)** The effect of different BMI levels on freshmen PFI. Note: when comparing male and female groups, ** (*p* < 0.01); A: BMI Low group; B: Normal group; C: Overweight group; D: Obesity group, multiple comparisons in one-way multivariate ANOVA when *p* < 0.05: A vs. B ω; A vs. C φ; A vs. D λ; B vs. C ε; B vs. D δ; C vs. D γ.

Figure 1 illustrates that various BMI classifications significantly (*p* < 0.05) impacted the outcomes of physical fitness assessments for both male and female students. The changes observed in the average performance of male and female students were similar, the change in performance of female students linked with BMI was less significant compared to male students concerning the 50 m sprint, mean pace of endurance run, standing long jump, core strength score and PFI score. In the male group, the low weight group demonstrated lower mean values of vital capacity, sit and reach, endurance running speed, and PFI in comparison to the normal weight group. Conversely, the low weight group showed higher mean values of 50 m sprint, standing long jump, 1,000 m run, and pull-up when compared to the normal weight group, with statistically significant differences observed in vital capacity, sit and reach (*p* < 0.01), standing long jump, and pull-up scores (*p* < 0.05). The differences in vital capacity, sit and reach (*p* < 0.05), and pull-up scores (*p* < 0.05) were particularly noteworthy (*p* < 0.05). Among male students categorized as super weight and obese, the mean values for vital capacity, 50 m sprint, sit and reach, and 1,000 m run were higher in the over-weight and obese groups than in the normal weight group. In contrast, the mean values for pull-up and standing long jump were lower in the overweight and obese groups. The differences in the overweight group were statistically significant, as were the differences in the obese group, except for the sit and reach (*p* < 0.01); When comparing females of different BMI groups, the average values for vital capacity, 50 m sprint, and sit and reach were lower than those of the normal weight group, with the mean values for pull-up and standing long jumps also being lower compared to the normal group. The mean values for flexion were significantly lower in the overweight and obese groups compared to the normal weight group (*p* < 0.01). Conversely, the mean values for vital capacity, 50 m sprint, and 800 m run speed were significantly higher in the overweight and obese groups than in the normal weight group. The mean values for 1 min sit-up were significantly lower in the overweight and obese groups compared to the normal weight group (*p* < 0.01). Furthermore, the differences in mean values for vital capacity and 800 m run speed were statistically significant (*p* < 0.01). Under different BMI groupings, both male and female students displayed the most favorable PFI indices within the normal weight group.

Simultaneously, the physical fitness indexes of male and female students in different BMI groups were analyzed. The results were combined using two-way ANOVA to examine gender differences. The statistically significant differences in vital capacity, standing long jump and pull-up indexes were observed among males in each group (*p* < 0.001), with significant differences in vital capacity indexes observed in females in each group (*p* < 0.001). The physical fitness indexes mean scores varied differently with BMI when comparing within the same gender group. Specifically, the vital capacity and standing long jump scores exhibited significant variability within the group, with BMI-related changes having a greater impact on their scores, whereas the other indicators had smaller differences between the low and normal weight groups. Statistically significant gender differences (*p* < 0.05) were observed at all BMI levels, except for the PFI scores in the low body mass group and the obese group. This finding suggests that the interaction effect of gender and BMI categorization exerts a greater influence on physical fitness test performance. The evaluation of the PFI that females with normal body mass scores had significantly higher scores than males (*p* < 0.001), while males in the super obese group scored significantly lower than girls (*p* < 0.001). Females (*p* < 0.001) showed no significant difference in BMI changes among the four groups, while for males, the impact of BMI changes is significant (*p* < 0.05). The difference between the four groups of male students is statistically significant. The ANOVA results indicate that BMI had a significant main effect (*p* < 0.001) on all physical fitness measures, with the exception of endurance running (η^2^ = 0.068) which exhibited a medium effect size, and the rest had small effect sizes (η^2^ < 0.06). Moreover, gender contributed significantly to the observed results (*p* < 0.05), with vital capacity, 50 m sprint, and standing long jump recorded large effect sizes (η^2^ ≥ 0.14). All interaction effects were significant (*p* < 0.001), although all had small effect sizes.

**FIGURE 2:**
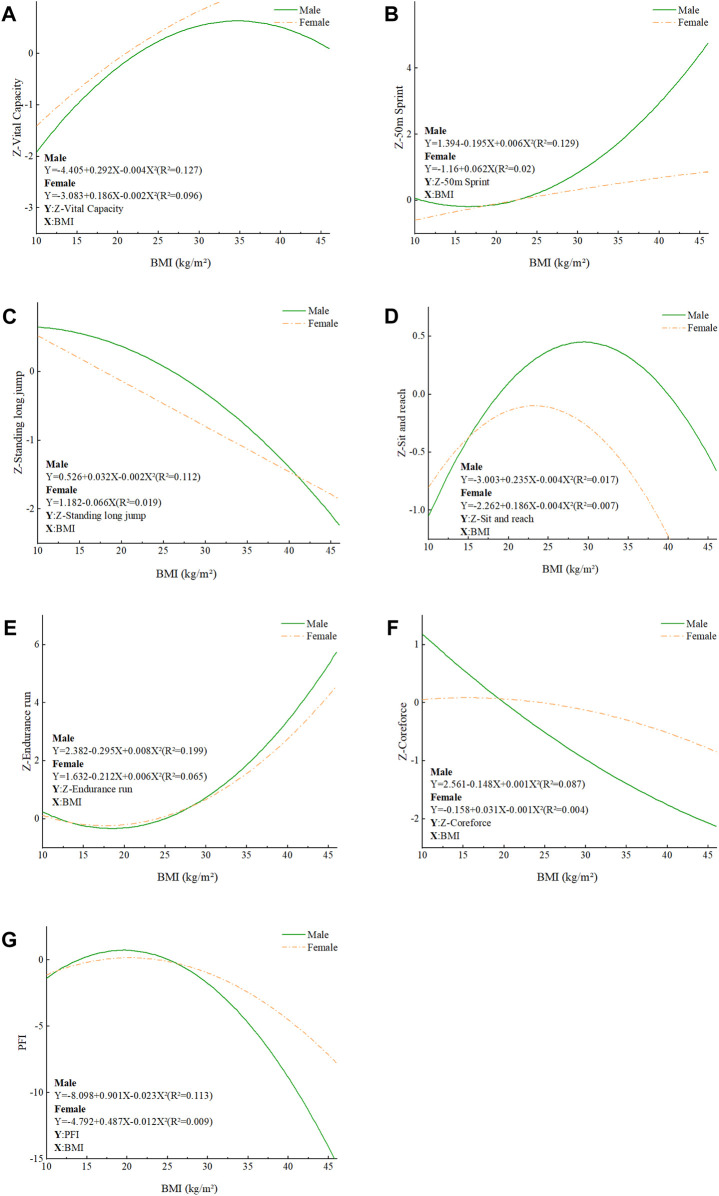
Relationship between BMI and Z-scores of various physical fitness tests among students of different genders. **(A)** Relationship between BMI and Z-Vital capacity in students of different genders; **(B)** Relationship between BMI and Z-50 m sprint in students of different genders; **(C)** Relationship between BMI and Z-standing long jump in students of different genders; **(D)** Rela-tionship between BMI and Z-sit and reach in students of different genders; **(E)** Relationship between BMI and Z-endurance run in students of different genders; **(F)** Relationship between BMI and Z-coreforce in students of different genders; **(G)** Relationship between BMI and PFI in students of different genders.

The correlation between each BMI category and Z-scores on the six health tests by gender is illustrated in Figure 2. Generally, an inverted U-shaped pattern was observed in vital capacity (*R* ^2^ of 0.096–0.127), male students’ standing long jump (*R* ^2^ of 0.112), sit and reach (*R* ^2^ of 0.007–0.017), and female students’ sit-up (*R* ^2^ of 0.004), implying that the highest values were noted in individuals of normal-weight group. For the 50 m run (*R* ^2^ ranging from 0.02 to 0.129) and the male endurance run (*R* ^2^ ranging from 0.065 to 0.199), the correlation showed a U-shaped, which implies that the normal BMI group has the highest score, followed by the low-weight group (Prista et al., 2003), while the overweight and obese groups have lower scores. In males’ pull-up (*R* ^2^ of 0.087) and females’ standing long jump (*R* ^2^ of 0.019), the straight line is approximately high and low on the left and low on the right. With the increase of BMI, the performance gradually worsens in a linear way. Students with normal BMI scored higher in physical fitness tests and showed more obvious performance in male regression relationship than female students (Du et al., 2017).

With PFI as the dependent variable and BMI as the independent variable, one-dimensional quadratic regression analysis was conducted. The results showed that the regression equations of male and female students were: PFI = −8.098 + 0.901BMI-0.023BMI^2^ (*R* ^2^ = 0.113, F = 295.879, *p* < 0.001) and PFI = −4.792 + 0.487BMI-0.012BMI^2^ (*R* ^2^ = 0.009, F = 20.018, *p* < 0.001); When males BMI is 19.59 and females BMI is 20.29, the PFI values are 4.03 and 0.15, respectively, which are the maximum values. When males BMI is close to their normal weight, the PFI values of males and females BMI are at a higher level.

The graph of the relationship between different BMI and PFI also shows that the trend line is parabolic regardless of gender, and that the trend is more pronounced in males than in females, indicating that the PFI of males with normal BMI is significantly better than that of the overweight and obese group, and the gap is even smaller in the female students’ group. At the lower end of the BMI range, the PFI increases linearly with the increase in BMI until the middle range of BMI, where the increase slows down, and the continued increase in BMI beyond the extreme point causes a significant decrease in PFI, which is also consistent with the findings of HUANG et al. (Huang and Malina, 2007; Bi et al., 2019).

## 4 Discussion

This study aimed to explore the relationship between BMI and physical fitness among university freshmen of different genders by conducting a physical fitness test on students enrolled in a university in Beijing, China, from 2018 to 2021.

Judging from the difference of freshmen in different enrollment years, the mean values of BMI for freshmen of different genders shows an increasing trend (Grasdalsmoen et al., 2019) and will reach the maximum in 2020, which is also consistent with the results of some studies (Dong et al., 2023; Hu et al., 2022; Han et al., 2023; Sun et al., 2023; Feng et al., 2023; Zhao et al., 2022), which may be due to the lockdown during the epidemic and changes in dietary habits (Touyz et al., 2020). Increased sedentary behavior among students and lack of freedom to exercise (Kerkadi et al., 2019; Tavolacci et al., 2021). The mean value of vital capacity reaches the highest value in the class 2020, which is consistent with the positive correlation with BMI (Wu, 2022). For adults, the heavier the weight, the greater the vital capacity, but the vital capacity is also affected by age, gender, height, weight, chest circumference and other factors (Zhang H. et al., 2022). However, it did not maintain an upward trend in the class of 2021, which is contrary to the findings of Sun et al. (2023), whose study indicated that the students’ vital capacity continued to increase in 2021. In addition, the scores of all other physical fitness indicators almost reached the lowest in grade of 2020, and in general, the scores of most physical fitness indicators of freshmen in the grade 2021 improved compared with those of freshmen of grade 19 and 20, and approached or even exceeded the scores of freshmen of grade 18 in some projects, which may be related to the resumption of offline teaching in most high schools and universities (Sun et al., 2023). This also shows that offline physical education courses can regulate students’ physical activities more effectively than online physical education courses, and it is also more conducive to improving their physical quality. On the ground of the setting of school physical education curriculum, the amount of time students spend participating in classroom and after-school physical activities is on the increase. The orderly opening of sports facilities will also increase the opportunities for after-school physical activity, and also facilitate the extension of students’ physical education time due to the health risk warnings during the epidemic (Chen P. et al., 2020), which made the community, schools, families, and students themselves pay more attention to their physical health.

Despite the data collection ending in 2021, the observed trends and relationships remain relevant today. Recent studies indicate that the issues of obesity and physical inactivity continue to be prevalent among college students (Han et al., 2023; Maitiniyazi et al., 2021; Sun et al., 2022), highlighting the ongoing need for effective physical education programs. Future research should consider updating the dataset to include more recent years, ensuring the trends and recommendations remain aligned with current conditions. Additionally, exploring the impact of new health initiatives and changes in lifestyle post-pandemic could shed light on further understanding of how BMI and physical fitness are evolving.

In the post-pandemic era, human function declines rapidly during recovery, and high-intensity exercise may cause negative effects (Hallal et al., 2012). Existing studies (Ye et al., 2022; Wang et al., 2021; Zheng et al., 2018; Cheng, 2015) have confirmed that traditional Chinese qigong exercises such as Baduanjin are beneficial for improving students’ physical performance, improving their vital capacity, flexibility, endurance running and body composition, improving students’ physical movement function and promote their coordinated physical and mental development, and reducing the risk of chronic diseases in people with various health conditions. Baduanjin is a widely used traditional Chinese qigong exercise method characterized by gentle and consistent postures, abdominal breathing coordinated with movement, and a meditative state of mind, which integrates the body and spirit, a strength of many traditional qigong exercises (Zou et al., 2018). Traditional qigong (Chan et al., 2019) such as Baduanjin are low-intensity and less difficult to learn, and can be easily promoted in school classes or on communication platforms such as we media.

It was also found that females are more concerned about their weight than males, so they have a much higher percentage of normal weight than males, and significantly lower rates of obesity and overweight than males. A study (Lee et al., 2021) conducted in the United States discovered that students’ BMI is influenced by peer pressure and environment. Females, influenced by their roommates, expressed a greater willingness to lose weight compared to male. This could be due to their anxiety about appearance and body image, and the fact that females are more likely to feel weight discrimination, prompting them to make more efforts towards weight loss (Yaemsiri et al., 2011; Harring et al., 2010; Roehling et al., 2007). Although most of the function and quality indicators of low weight students are better than obese and overweight students, but in the absolute indicators such as vital capacity and sit-and-reach test scores are worse, which suggests that the body type is too thin will also have a certain negative impact on the physiological function and quality of college students, for example, female students due to irrational diet control, the lack of necessary physical activity (especially strength training), may lead to low BMI to a certain extent, and the loss of muscle strength, osteoporosis, and endocrine disorders caused by low body weight may take their toll on the physical health of college students. The study also highlighted the impact of long-term roommate connections. During their college careers, roommates have a significant influence on each other’s weight over time, as they spend the most time together as peers. Utilizing mechanisms of individual interaction can prove advantageous in preventing obesity (Liu et al., 2021). BMI is not just correlated with fitness, but also with the risk of injury during physical education classes (Onea and Balint, 2018). One study discovered that male students who were overweight or obese had a greater likelihood of injury compared to female students of the same grade level during the evaluated academic year, and for self-perception, the opposite was evident (Morano et al., 2011). Female students had higher levels of body dissatisfaction than their male counterparts, and overweight or obese students reported higher levels of body dissatisfaction than their peers with a normal weight. Furthermore, obesity had a harmful impact on athletic performance as well as body self-perception. Therefore, weight interventions for college students should not only target obese and overweight students, but also pay special attention to low weight students.

Differences in student BMI differentially affected flexibility (sit-and-reach test, cardiorespiratory fitness (vital capacity and endurance running), explosive strength (standing long jump and 50 m sprint), and strength endurance (sit-up and pull-up).

(1) The best performance in flexibility-related indicators (sit-and-reach test) is seen in the super group of men, while the low body group shows the poorest performance. There was no statistical significance between the normal weight group and the obese group, and the differences among other groups were significant, the score difference between the low weight group and the other three groups was statistically significant, the obesity group had the best performance, and BMI was positively correlated with the performance of sitting forward flexor, being too thin may have a negative impact on the flexibility qualities of college students (Martinez-Lopez et al., 2019; Thompson et al., 2021; Chiang et al., 2022). Moreover, studies have shown that muscle flexibility has been proven to predict body fat obesity in subjects (Chiang et al., 2022). The physical education curriculum involved less specific training for flexibility, and the students who maintained normal weight participated in more physical activities, but there was a certain probability that they ignored the flexibility exercises, which also affects the students’ test results to a certain extent.
(2) Regarding cardiorespiratory function indices, spirometry scores for both male and female students increased with increasing BMI, possibly due to increased respiratory muscle strength in individuals with higher body mass, but spirometry performance still declined above a certain BMI value. This trend is supported by existing studies that evaluated the health indices of students using vital capacity index (VCI = VC (mL)/weight (kg)) and found that abnormal body weight negatively affected spirometry weight index performance (Chen X. et al., 2020; Zhang H. et al., 2022). Endurance running scores exhibited a general decrease, however, freshman in grade 21 outperformed their counterparts in grades 19 and 20. Several studies indicate that obesity adversely affects cardiorespiratory fitness (Wu, 2022; Beets et al., 2005; Hung et al., 2022), which is highly correlated with cardiovascular disease. It has been observed that physical activity and health-related fitness behaviors of college students commonly decrease, leading to weight gain in the college years, especially for those with higher BMI. It was also noted that students’ physical activity and health-related fitness behaviors show a downward trend during college years. This causes them to gain weight, while students with higher BMIs are less likely to improve in endurance running, muscle strength and endurance, which also has a negative impact on cardiorespiratory fitness. While the body’s ability to exercise is determined by the overall performance of the cardiovascular and respiratory systems, weight gain in college students also has a negative impact on their other athletic abilities. More colleges and universities carry out extra-curricular activities (Buckley and Lee, 2018) such as campus running, which has a good role in promoting students’ endurance and developing exercise habits (Li, 2022), reducing the obesity rate and reducing the possibility of suffering from chronic cardiovascular diseases.
(3) The standing long jump and the 50 m sprint are considered to be tests index for measuring physical explosive power. Under the same physical conditions, students with low BMI are more likely to achieve better results, which is negatively correlated with the change of BMI grade (Sacchetti et al., 2012), and is more significantly correlated with the performance of male students. The low body weight group of both male and female students had the best performance, and the difference was not statistically significant compared with the normal body weight group, but the difference was more significant compared with the overweight and obesity group. After subdividing the low-weight group, (Cai et al., 2010) found that too low body weight may not be conducive to achieving the best performance in the standing long jump, possibly because the muscle content in the low-weight group also decreases, and the absolute strength also decreases accordingly. It has been noted that an increase in BMI may reflect an increase in fat or muscle (Dollman et al., 1999). Kidokoro found that there was a significant improvement in students’ BMI when grip strength was significantly reduced, suggesting that there may be a significant decrease in unit muscle strength (Kidokoro et al., 2020). The lack of lean body mass and other test indicators to evaluate the body fat percentage of students in the physical fitness test (Alves Junior et al., 2017), and the division of students by BMI only may lead to a certain deviation in the prediction of strength performance.
(4) In terms of strength endurance, in the sit-up test of female, the normal-weight group had the highest score, and there was no statistically significant difference between them and the low-weight group. The low weight group of male pull-up had the best performance, followed by the normal group. In both sexes, the low weight group and the normal weight group had significantly better performance than the overweight and obese group. The low weight group was more conducive to pulling the body because of its lower self-weight (Wu, 2022), and when the weight increased, the demand for strength also doubled, which was unfavorable to the heavier people (Kwok-Kei Mak et al., 2010). Students without strength training have a harder time doing this. In the regression equation, female students showed an inverted U-shape while male students showed a U-shape curve. With the increase of BMI, female students had the best core strength endurance performance in the normal weight range, while male students’ upper body strength endurance performance decreased, and the decline speed accelerated when approaching overweight. BMI has a greater impact on strength endurance, which may be more because weight gain also increases the difficulty of movement completion.

Since freshmen still have to spend most of their time on study and may lack time for physical activity, multiple studies have pointed out that school-based physical education classes still play a major role in improving students’ BMI and PFI (Bao et al., 2020). Aerobic exercise (Yang et al., 2012), functional fitness training (Lou and Fu, 2023) or other sports such as ball games (Lee et al., 2021) have a positive impact on students’ physical health, improve students’ physical fitness, assist in weight loss, and reduce the risk of chronic diseases.

It should be noted that when discussing the relationship between physical activity and physical health, we should not only focus on the physical health of students, but also consider the coordinated development of their psychology. The improvement of physical activity level and exercise behavior will play a positive role in controlling BMI, but excessive and vigorous physical activity may reduce students’ self-reported mental health index (Han et al., 2023; Snedden et al., 2019). In recent years, the theme of fitness and weight loss has gradually increased on Chinese social media, and the public has a higher pursuit of physical health and a perfect body. People usually have weight bias towards obese people (Wu, 2022), and studies have shown that these people are more likely to have psychological problems, especially in adolescence. We should pay attention to students’ mental health while paying attention to their physical health (Bao et al., 2020).

We also acknowledge that there are some limitations to this study. Firstly, the research object is freshmen in a university, which may not be representative in a broad sense and lacks longitudinal comparison design. Secondly, there is a lack of investigation on students’ psychological status, after-school sports, lifestyle and other influencing factors. In addition to physical activity, behavioral, social and environmental factors (Lu et al., 2022) are likely to significantly affect their health status. Future research should include a longitudinal design and a more diverse population to improve generalizability. Additionally, incorporating psychological wellbeing assessments could provide a more holistic view of student health. Moreover, the study was conducted using data from 2018 to 2021, a period which included significant lifestyle changes due to the COVID-19 pandemic. While this provides valuable insights into the impact of the pandemic on physical fitness, it also means that the results may not fully represent trends before or after this period. Future studies should consider examining data over a longer timeframe to provide a more comprehensive understanding of these trends. Future studies can carry out more perfect statistics and investigations, which will be more conducive to clearly analyzing students’ physical changes and making corresponding suggestions.

## 5 Conclusion

In conclusion, Body Mass Index (BMI) has different effects on various physical qualities of freshmen. A Normal BMI level is the premise and basis for ensuring students’ physical health. Low weight significantly impacts freshmen’ cardiopulmonary endurance and flexibility, while overweight and obesity are important factors affecting students’ physical health, which seriously limits the development of freshmen’ physical qualities such as explosive power and endurance. And it increases the risk of sports injuries.

Consequently, physical education in colleges and universities should strengthen the accurate monitoring and intervention of the BMI level of freshmen. It is important to improve students’ understanding of the significance of achieving and maintaining a normal BMI level, effectively reducing the overall overweight and obesity rate among school students. Additionally, there should be an increase in strength, speed, flexibility, and other specific training modules to promote the overall physical and mental health development of students.

Simultaneously, for low weight and overweight people, we can infer their physical and physical deficiencies according to their BMI and other body composition test indicators. Targeted physical education courses should be arranged upon entering school to effectively improve their physical performance and promote physical health.

In summary, recognizing the intricate relationship between BMI and physical fitness in college freshmen is crucial. Addressing these factors proactively through targeted interventions and holistic physical education programs will contribute to the comprehensive development of students’ physical and mental health.

## Data Availability

The data used to support the findings of this study are available from the corresponding author upon request. Requests to access these datasets should be directed to Tongtong Guo, gtt9811@163.com.
